# Influence of the anti‐oestrogens tamoxifen and letrozole on thyroid function in women with early and advanced breast cancer: A systematic review

**DOI:** 10.1002/cam4.4949

**Published:** 2022-06-24

**Authors:** Djordje Marina, Åse Krogh Rasmussen, Kristian Buch‐Larsen, Linn Gillberg, Michael Andersson, Peter Schwarz

**Affiliations:** ^1^ Department of Endocrinology and Metabolism, Rigshospitalet Copenhagen Denmark; ^2^ Department of Biomedical Sciences, Center for Healthy Aging Copenhagen University of Copenhagen Copenhagen Denmark; ^3^ Department of Oncology, Centre for Cancer and Organ Diseases, Rigshospitalet Copenhagen Denmark; ^4^ Faculty og Health and Medical Sciences University of Copenhagen Copenhagen Denmark

**Keywords:** breast cancer, letrozole, subclinical hypothyroidism, tamoxifen, thyroid hormones, weight gain

## Abstract

**Introduction:**

Breast cancer (BC) is a common type of cancer in women. Advances in therapy options have resulted in higher overall survival rates but side effects of cancer treatment are increasingly in the spotlight. The beneficial effects of anti‐oestrogen therapy with tamoxifen and letrozole in the prevention of BC recurrence are well documented. While the most common side‐effects of this therapy are well‐defined, less is known about its effects on thyroid function. In women treated for early BC, an average of 1–5 kg weight gain has been observed after treatment with chemotherapy/anti‐oestrogens. We aim to evaluate the current knowledge on the side effects of tamoxifen and letrozole treatments on thyroid function, followed by its potential influence on the observed weight gain.

**Methods:**

We searched PubMed and found 16 publications on thyroid function and tamoxifen treatment in pre‐ and post‐menopausal women with early‐ and advanced BC, whereas five publications on letrozole treatment in post‐menopausal women with advanced BC.

**Results:**

According to the current literature, there is an overall tendency towards a mild and transient thyroid dysfunction, that is, subclinical hypothyroidism in tamoxifen‐treated patients. Only one publication reported further significant changes in thyroid hormones beyond one year of tamoxifen treatment. No significant changes in thyroid function have been observed among letrozole‐treated patients.

**Conclusion:**

Tamoxifen‐treated patients can develop mild and transient thyroid dysfunction within the first 12 months, yet further significant changes in thyroid function beyond one year of tamoxifen treatment have been reported in a single study. There is no evidence of thyroid dysfunction in letrozole‐treated patients. Current literature does not focus on subclinical hypothyroidism as a possible cause of weight gain in patients with BC. Subgrouping of BC patients and studies with a longer observation of thyroid hormones and weight changes during and after anti‐oestrogen treatment are needed to further elucidate how anti‐oestrogens affect thyroid function.

## Graphical abstract

Although anti‐oestrogens tamoxifen and letrozole have been used for more than a decade, less is known about the side effects of these treatments on thyroid function. Breast cancer patients gain on average 1‐5 kg during the treatment with chemotherapy/anti‐oestrogens, yet for unknown reasons. We performed a systematic review to see if tamoxifen/letrozole can influence thyroid function and thereby can partly explain weight gain in patients treated for breast cancer.

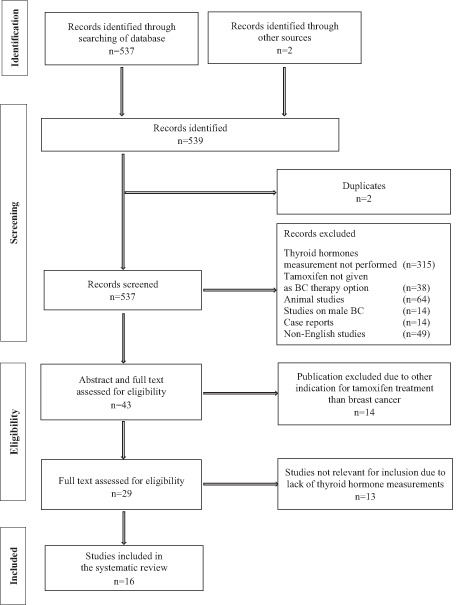

## 1 INTRODUCTION

Breast cancer (BC) is commonly diagnosed, being the fifth cause of cancer deaths in the world.^1^ The incidence rate of BC is rising, probably reflecting the increased prevalence of risk factors, early detection by mammography screening, and aging,^2^ while the mortality rate is decreasing, suggesting substantial advances in cancer therapy.^3^ Overall survival of patients with BC is still the primary goal, but unintended side effects of BC treatments are gaining increasing attention.^4^, ^5^ Breast cancer treatment is dependent on disease staging, menopausal status, and receptor type in BC cells (oestrogen‐positive cells, human epidermal growth factor receptor 2 [HER2]‐positive cells, or progesterone‐positive cells). Therapy options can include: Breast surgery, pre‐ or postoperative chemotherapy, local or locoregional radiotherapy, adjuvant anti‐oestrogen therapy with tamoxifen or aromatase inhibitors, or therapy with HER2‐inhibitors.^6^ Approximately 75% of all BC patients have oestrogen‐receptor‐positive cancer, which is predictive of the effect of antihormonal therapy.^7^ Since patients diagnosed with early‐stage BC are at risk of long‐term cancer recurrence (>5 years after the diagnosis), prevention treatment with anti‐oestrogens is indicated to decrease the risk of recurrence and improve BC‐specific survival.^8^

Main adjuvant treatments include tamoxifen (selective oestrogen receptor modulator [SERM]) and aromatase inhibitors (AIs). Both medications lower the risk of BC recurrence by reducing the effect of oestrogen on breast cancer cells, yet in different ways. Tamoxifen is the oldest and widely used non‐steroid anti‐oestrogen. Tamoxifen acts in the tissue‐specific agonist–antagonist manner: It has anti‐oestrogenic effects in breast tissue and on the receptors of BC cells, yet oestrogenic, partial agonistic effects on bones, cardiovascular system, and endometrium.^9^ Although originally introduced as a treatment for advanced BC in post‐menopausal women, tamoxifen is now preferred in pre‐menopausal women with early BC.^10^ The newer anti‐oestrogen treatment (i.e., AIs such as letrozole, anastrozole, and exemestane) has been recommended in post‐menopausal women with early‐stage (non‐metastatic)^11^, ^12^ and advanced (metastatic) BC,^13^ with beneficial results. Letrozole inhibits the enzyme aromatase by competitive binding to the cytochrome P450 subunit of the enzyme, which results in a reduction of oestrogen synthesis in all tissues. Letrozole treatment significantly lowers serum oestrone, oestradiol, and oestrone sulfate. A randomised controlled trial on oestrogen‐suppressive effects of letrozole versus anastrozole showed letrozole is a more potent AI.^14^ When AI is not tolerated, tamoxifen can still be effectively used.

Adjuvant BC therapy can cause several side effects, mostly related to the loss of oestrogen effect. Yet, in most patients, the benefits far outweigh the risk of side effects, which is why tamoxifen and AI are a cornerstone of BC adjuvant therapy. Already in the late 1970s, it was described that women gain on average 3–5 kg after the chemotherapy, yet for unknown reasons.^15^, ^16^, ^17^ Recently, Buch‐Larsen et al. showed an average weight gain of 1.2 kg after the chemotherapy.^18^ Furthermore, anti‐oestrogens have been demonstrated to induce weight gain, with conflicting results. Tamoxifen has been documented to influence weight gain in some,^19^, ^20^ but not all studies,^21^, ^22^ whereas anastrozole seems not to increase the risk of weight gain.^21^ In patients with BC, many factors, such as metabolic changes, metabolic rate, physical activity, and changes in eating patterns due to physiological disturbances related to diagnosis might be the possible explanation for weight gain,^21^ but they are not yet scientifically confirmed.

As thyroid dysfunction (i.e., hypothyroidism) and weight gain are closely related it can be hypothesized that anti‐oestrogens could influence thyroid function and contribute to the observed weight gain. Oestrogen has an indirect and direct effect on the thyroid gland. Indirectly, oestrogen increases the concentration of thyroxine‐binding globulin (TBG), causing variations in the total thyroxine (TT4), and total triiodothyronine (TT3) without changes in free‐thyroxine (FT4) and free‐triiodothyronine (FT3).^23^ Directly, oestrogen influences oestrogen‐receptors, being described in both neoplastic and non‐neoplastic human thyroid tissue, which can modulate the proliferation and function of thyroid cells.^24^ Furthermore, aging is a risk for an increase of thyroid‐stimulating hormone (TSH) in people without known autoimmune thyroid disease, probably influenced by changes in the TSH set‐point.^25^

It is still speculative whether patients with BC have a higher incidence of thyroid dysfunction or the degree to which different BC treatments (radio‐, chemo‐ and anti‐oestrogen therapy) alter thyroid function. Some observational studies have suggested that BC patients may have a higher risk of developing hypothyroidism per se, probably most influenced by radiotherapy, as documented in some─but not all─studies.^26^, ^27^, ^28^ Meanwhile, the scientific literature on the influence of anti‐oestrogen therapy on thyroid function is sparse.

The purpose of this systematic review is to evaluate our current knowledge of thyroid function concerning the two most widely used anti‐oestrogen treatments for BC (tamoxifen and letrozole) to elucidate if thyroid dysfunction might be more frequent and hence a contributing factor to observed weight gain in patients with BC.

## 2 METHODS

### 2.1 Study design

This systematic review has been conducted and presented following the PRISMA (Preferred Reporting Items for Systematic review and Meta‐analyses) statement recommendations and was registered at PROSPERO (registration number: CRD42021261098).

We aimed to evaluate whether the current literature shows evidence of thyroid dysfunction in BC patients treated with tamoxifen or letrozole as well as if the possible changes could influence the weight gain of these patients.

### 2.2 Search strategy and study selection

Two authors (DJM and KB‐L) performed an independent literature search on PubMed. Initially, a broad search included two SERMs (tamoxifen and raloxifene), three AIs (exemestane, anastrozole, letrozole), fulvestrant (selective oestrogen receptor degrader [SERD]), and each combined with thyroid function has been performed. However, no publications on raloxifene, anastrozole, and fulvestrant relating to thyroid function were found, yet only one case report on exemestane, which induced subclinical hypothyroidism, was found.^29^ Consequently, all articles describing the effect of adjuvant tamoxifen‐ and letrozole treatment on thyroid function in women with early‐ and advanced BC were identified and included. No limits to follow‐up time of thyroid hormone measurements or publication date were specified in an attempt to get all relevant articles during the first search. The following exclusion criteria were used: Non‐English papers, animal studies, studies on male BC, case reports, no access to full text, publications in which tamoxifen/letrozole were treatment options but no thyroid hormone data were given or medications were not used as the treatment of BC. English abstracts for foreign language publications were assessed to see if any relevant publication was overlooked. Screening of publications was initially performed by title reading, followed by a screening of abstracts to elucidate if thyroid function was a part of the paper. Finally, the articles selected were evaluated by full‐text reading.

We included BC patients in both early‐stage, advanced stage (metastatic/locally advanced), and with unknown status. More selective search terms regarding BC–staging have given a very limited number of articles, which were not relevant for inclusion in the systematic review. For this reason, the BC type has been removed from the search engine.

We performed the final PubMed search on the 16th of April 2021 with the above‐named exclusions and with the following search terms related to tamoxifen:

(tamoxifen OR tamoxifen citrate OR nolvadex OR soltamox)

AND

(thyroid OR thyroid hormones OR thyroid function OR thyroid disease OR thyroid gland OR TSH OR FT4 OR T4 OR FT3 OR T3).

We performed the final PubMed search on the 20th of May 2021 with the above‐named exclusions and with the following search terms related to letrozole:

(letrozole OR femara OR CGS 20267)

AND

breast cancer.

AND

postmenopausal patients.

AND

(thyroid OR thyroid hormones OR thyroid function OR thyroid disease OR thyroid gland OR hormones OR TSH OR FT4 OR T4 OR FT3 OR T3).

### 2.3 Literature search results on tamoxifen

**FIGURE 1:**
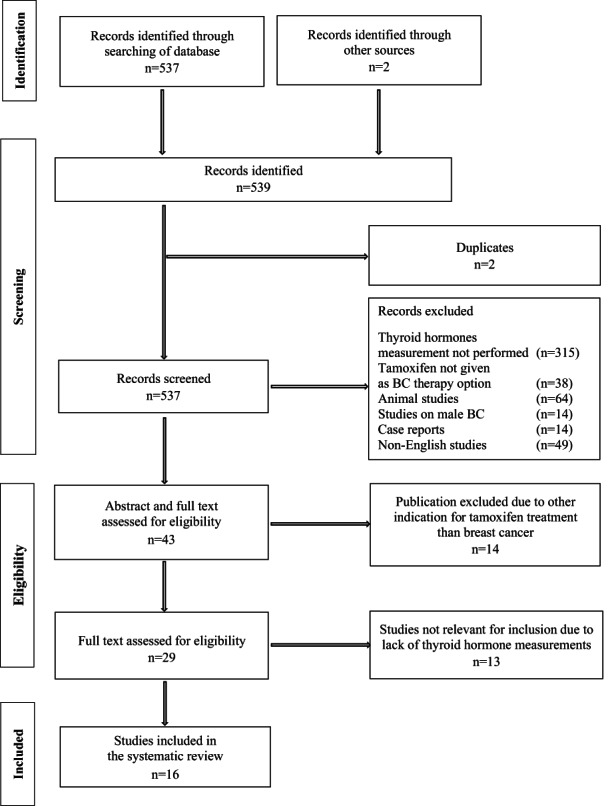
Inclusion of publications on tamoxifen and thyroid hormones.

We initially identified a total of 537 publications in the tamoxifen search and removed two duplicates. Furthermore, we found two relevant publications in the bibliographic sources of other research articles. These two publications did not appear during the search on PubMed, and the full text was accessed via Research Gate^30^ and Google Scholar^31^ due to their relevance for inclusion. All studies were screened for eligibility by reading the title and abstract, and a total of 494 publications were excluded due to (i) lack of thyroid hormone data or when BC was not the indication for tamoxifen treatment (*n* = 353), (ii) animal studies (*n* = 64), (iii) articles on male BC (*n* = 14), (iv) case reports (*n* = 14) and (v) non‐English articles (*n* = 49), leaving 43 publications for evaluation. Based on abstract and full‐text evaluation 14 studies were excluded due to other indications for tamoxifen treatment than BC, and we performed a full‐text evaluation of 29 papers. Thirteen publications were excluded due to lack of thyroid hormone measurements, whereas 16 publications on tamoxifen evaluated thyroid hormones were eligible for inclusion in the systematic review.^25^, ^26^, ^27^, ^28^, ^29^, ^30^, ^31^, ^32^, ^33^, ^34^, ^35^, ^36^, ^37^, ^38^, ^39^, ^40^ A detailed flow diagram is shown in Figure 1.

### 2.4 Literature search results on letrozole

**FIGURE 2:**
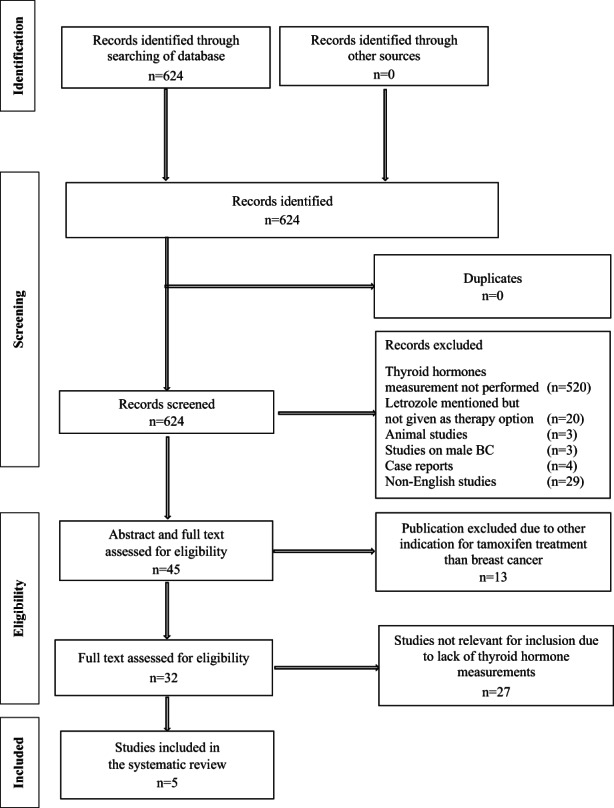
Inclusion of publications on letrozole and thyroid hormones.

Our literature search of letrozole‐treated BC patients and thyroid function resulted in 624 publications. We did not find any duplicates. All studies were screened for eligibility and a total of 579 publications were excluded due to (i) lack of thyroid hormone data or when letrozole was mentioned but not given as the treatment option (*n* = 540), (ii) animal studies (*n* = 3), (iii) articles on male BC (*n* = 3), (iv) case reports (*n* = 4) and (v) non‐English articles (*n* = 29), leaving 45 publications for evaluation. Based on abstract and full‐text evaluation 13 studies were excluded due to other indications for letrozole treatment than BC, and we performed a full‐text evaluation of 32 papers. This evaluation showed that 27 studies were not eligible for inclusion due to a lack of thyroid hormone measurements. At last, five studies on aromatase inhibitors, all using letrozole, entered the current systematic review.^46^, ^47^, ^48^, ^49^, ^50^ A detailed flow diagram is shown in Figure 2.

### 2.5 Risk of bias assessment

Two investigators (DJM and KB‐L) independently assessed the included studies for risk of bias using the ROBINS‐I tool.^51^ Overall, the studies showed a low to moderate risk of bias. Results of the risk of bias assessment are visualized by the Risk‐Of‐Bias VISualization tool (robvis)^52^ and summarized in Table S1 for tamoxifen studies, and in Table S2 for letrozole studies.

### 2.6 Patient characteristics and results in the tamoxifen group

Sixteen studies on thyroid function related to tamoxifen treatment were included in this systematic review: 124 pre‐menopausal‐ and 451 post‐menopausal women in different stages of BC. One publication described a group of 45 women with BC, in whom the menopausal status was not known.^44^ Thirteen of the included studies were performed as cohort studies and three as randomised clinical trials. Further information about these studies is shown in Table 1.

**TABLE 1:** Publications on tamoxifen and thyroid function included in the systematic review

Publication	Study design	Breast cancer stage	Mean/median age of patients (years)	Pre‐menopausal women (*n*)	Post‐menopausal women (*n*)	Prior breast cancer treatment/number of patients (*n*)	Dosis of tamoxifen (mg/day)	Duration of tamoxifen (months)	Time of thyroid hormone evaluation	Hormone measurements	Observed changes in thyroid function	Type of thyroid hormone dysfunction
Anker GB et al.^32^	Observational cohort study	Unknown	Median 64	None	26	Locoregional radiotherapy (*n* = 8)	30	6	Baseline + monthly in 1 year	TSH FT4 FT3 TBGTG	Months 6–8: ↑TSH* ↓FT4** ↓FT3** ↑TBG*** ↑TG* Months 9–12: ↑TSH* ↓FT4* Months 13+: ↑TSH** ↓FT4*** ↓FT3** ↑TBG***	Months 6–8: Hypothyroidism Months 9–12: Hypothyroidism Months 13+: Hypothyroidism
Blackburn AM et al.^38^	Randomized‐controlled trial	Metastatic	Median 58	None	10	None	20	12	Baseline + months 1 + 6 + 12	FT4	→ FT4	None
Bruning PF et al.^42^	Observational cohort study	Locally advanced	Mean: pre‐menopausal 43.3 post‐menopausal 63.2	8	46	Unknown	10	6	Baseline + months 2 + 6	TSH TT4¤	Months 2 + 6 in both groups: ↑TSH** ↑TT4**	Subclinical hypothyroidism
Cuzick J et al.^43^	Observational case–control study	Unknown	Unknown	1	14	Unknown	20	Median 72	Once, after long‐term therapy	TT3 TT4	↑TT3** with 19% compared to controls ↑TT4** with 30% compared to controls	Undefined, lack of TSH measurement
Delrio G et al.^40^	Observational cohort study	Stage I‐II, operable	Median 59	10	60	Radical mastectomy	10	24	Baseline + at 3‐months intervals for 1 year	Basal TSH TRH‐stimulated TSH	→ both TSH measurements	None
Gordon D et al.^41^	Observational case–control study	Unknown	Unknown	None	10	None	20	Median 11	Baseline + week 1 + month 3	TSH TT4 FT4 TT3 TBG	↑TT4* (18% of patients) ↑TT3* (2% of patients) ↑TBG* (90% of patients)	None
Grani G et al.^36^	Observational case–control study	Unknown	Mean 59	Non‐ selected patients regarding menopause status (*n* = 45)	Non‐ selected patients regarding menopause status (*n* = 45)	Unknown	Unknown	60	After 5 years of therapy	TSH FT4 FT3	Hypothyroidism (8.9%) Hyperthyroidism (4.4%) Chronic autoimmune thyroiditis (24.4%)	Hypothyroidism (8.9%) Hyperthyroidism (4.4%) Chronic autoimmune thyroiditis (24.4%)
Kim H et al.^37^	Observational cohort study	Ductal carcinoma in situ	Median 42	36	23	Radiotherapy: patients with clinical amenorrhoea (*n* = 17) post‐menopausal patients (*n* = 16)	20	Median 24	Between cycle day 2–5 (pre‐menopausal patients) Single measurement (patients with amenorrhoea)	TSH	↓TSH** (amenorrhoea patients) →Mean TSH (both groups)	Subclinical hyperthyroidism
Kostoglou‐Athanassiou I et al.^45^	Observational case–control study	Non‐metastatic	Mean 62.4	None	42	Breast cancer surgery	20	6	Baseline + month 6	TSH FT4 TT4 FT3 TT3 TBG	↑TSH** ↑TT4** ↑TT3*** ↑TBG*** →FT3 →FT4	Subclinical hyporthyroidism
Mamby CC et al.^44^	Randomized‐controlled trial	Non‐metastatic	< 65 (range 46–64)	None	14	Unknown	20	3	Baseline + month 3	TSH TT4 TBG Calculated FTI	↑TT4*** ↑TBG***	None
Michalaki V et al.^35^	Observational case–control study	Invasive ductal, lobular, and mixed carcinoma (97.6%) Ductal carcinoma in situ (2.4%)	Median 63	58	85	Chemotherapy (*n* = 53) Chemotherapy + radiotherapy (*n* = 48)	Unknown	Unknown	Unknown	TSH FT4 FT3	→TSH →FT4 →FT3	None
Mirzaei HR et al.^31^	Non‐randomized uncontrolled study	Unknown	Age range 30–68	11	12	Chemotherapy before tamoxifen (78% of patients) Locoregional radiotherapy (*n* = 15)	20	3	Baseline + month 3	TSH FT4 TT3	↑TT3** ↑TSH* ↑FT4*	Subclinical hypothyroidism
Pannuti F et al.^30^	Observational cohort study	Locally advanced	Mean 61	None	13	Medroxyprogesterone acetate 2 months before (*n* = 2)	20	1	Baseline + month 1	TSH TT4 TT3	↓TT4** ↓TT3* ↑TSH*	Subclinical hypothyroidism
Panutti F et al.^39^	Randomized‐controlled trial	Locally advanced	Median 57	None	10	Chemotherapy (at least 1 month before) (*n* = 2)	20	1	Baseline + month 1	TSH TT4 TT3 FT4 FT3	↓TT4** →TSH →TT3 →FT4 →FT3	None
Zidan J, Rubenstein W.^33^	Observational cohort study	Stage 1 (*n* = 25) Stage 2 (*n* = 20)	Mean 65	None	45	None	20	Mean 19	Baseline + months 3 + 6	TSH FT4 TT3	Month 3: ↑TSH** ↓TT3** →FT4 Month 6: →TSH →FT4	Month 3: Subclinical hypothyroidism Month 6: None
Zidan J et al.^34^	Observational cohort study	Non‐metastatic	Mean 63.4	None	41	Mastectomy/ lumpectomy + dissection of axillary lymph nodes	20	Mean 19	Baseline + months 3 + 12	TSH FT4	Month 3: ↑TSH** →FT4 Month 12: TSH normalized →FT4	Month 3: Subclinical hypothyroidism Month 12: None

*Note*: →: within reference range; ↑: above upper limit of normal; ↓: below lower limit of normal.

Abbreviations: FT3, free triiodothyronine; FT4, free thyroxine; FTI, Free Thyroid Index; TBG, thyroxine‐binding globulin; TG, thyroglobulin; TRH, thyrotropin‐releasing hormone; TSH, thyroid stimulating hormone; TT3, total triiodothyronine; TT4, total thyroxine.

* Statistically non‐significant (*p* > 0.05).

** Statistically significant (*p* < 0.05).

*** Statistically highly significant (*p* < 0.001).

The studies were published from 1985 to 2017. The median/mean age of the patients ranged from 41 to 77 years (Table 1). The median duration of tamoxifen therapy was 17.8 months, with exception of Michalaki et al., where the treatment duration was not specified.^43^ The median tamoxifen dosage was 20 mg/day (range: 10–30 mg/day), yet two studies did not specify the dosage of tamoxifen^43^, ^44^ (Table 1). The time of thyroid hormones evaluation was in the median of 6 months after tamoxifen treatment, whereas this was not specified in the two studies.^37^, ^43^ Most studies have reported TSH, FT4, and FT3, and some studies reported TT4 and TT3 (Table 1). Yet, in two studies only TSH analysis was performed.^34^, ^45^ These studies were included, since TSH is still the most sensitive marker of thyroid disease, as documented in a recent review.^53^ Only two studies on tamoxifen mentioned weight gain.^36^, ^41^

Changes in thyroid‐stimulating hormone: TSH was measured in 14 of the 16 studies. In 13 studies, the follow‐up time after treatment initiation was up to 12 months. In one study TSH was measured after 12 months of treatment.^40^ Five studies showed a significant increase in TSH at months 2–12 after tamoxifen initiation.^36^, ^39^, ^40^, ^41^, ^42^ In BC patients with a mean cancer duration of 10.33 years (BC diagnosed 0–35 years in advance), Grani et al. have found that 8.9% of the patients have hypothyroidism and 4.4% have hyperthyroidism after 5years tamoxifen treatment.^44^ Yet, in both groups occurrence of thyroid peroxidase antibody (TPOAb) and TSH receptor autoantibodies (TRAb) was prevalent (24.4%), and mean TSH was significantly higher compared to the group of patients showing merely increased levels of serum autoantibodies.^44^ Two studies reported a trend of TSH elevation after 1 and 3 months of tamoxifen treatment, the latter one observed patients with subclinical hypothyroidism.^30^, ^31^ Kim H et al. studied 59 pre‐menopausal women with amenorrhoea and with normal menstruation after 2 years of tamoxifen treatment.^45^ Authors identified a significantly lower TSH in women with amenorrhoea (61% of the patients) compared to women with regular menstruation.^45^ Importantly, the prevalence of hypo‐ or hyperthyroidism was not different between these two patient groups.^45^ Only one study measured TSH beyond 12 months of tamoxifen therapy.^40^ Mean TSH at month 13+ was increased (highly significant) from baseline in 14 patients in contrast to the observed changes during the first 9 months of treatment (statistically significant TSH increase).^40^

Changes in total thyroxine/free thyroxine: TT4 was measured in 7 of 16 studies^30^, ^33^, ^35^, ^36^, ^37^, ^38^, ^39^ and FT4 was measured in 8 studies.^31^, ^32^, ^35^, ^39^, ^40^, ^41^, ^42^, ^43^ Follow‐up time for TT4 measurement after tamoxifen initiation was from 1 week to 6 months in all studies, except in one, where TT4 was measured after a median of 72 months (range 67–90) of tamoxifen treatment.^37^ Follow‐up time for measurement of FT4 was from 1 week to 12 months in most of the studies. However, Anker et al. further measured FT4 at 13+ months.^40^ Total thyroxine was shown to be significantly increased in 3 of 16 studies,^36^, ^38^, ^39^ as measured between months 2 and 6 after tamoxifen initiation. Furthermore, TT4 was 30% higher in tamoxifen users (median time of treatment 72 months, range 67–90 months) compared to the control group of ex‐tamoxifen users (median time off tamoxifen 58 months, range 47–74 months).^37^ Gordon et al. reported a non‐significant increase of TT4 in 18% of the patients.^35^ In two publications authored by Pannuti et al., TT4 significantly decreased 1 month after tamoxifen was initiated.^30^, ^33^ Measurement of FT4 was performed in 9 of 16 studies. In the majority (6 of 9 studies) no change in FT4 was observed in up to 12 months of follow‐up,^32^, ^35^, ^39^, ^41^, ^42^, ^43^ but it seems that FT4 is being furthermore significantly reduced as measured beyond 13 months of tamoxifen treatment.^40^ Again, the studies were with great variation. A statistically non‐significant increase in FT4 after 3 months was observed in the study of Mirzaei et al.^31^ and Grani et al. reported hypothyroidism in 8.9% and hyperthyroidism in 4.4% of the patients after the 5‐years of tamoxifen therapy.^44^ Yet, patients with positive TPOAb and TRAb were prevalent (24.4%).^44^

Changes in total triiodothyronine/free triiodothyronine: Assessment of TT3 was performed in 7 of 16 studies and FT3 in 5 of 16 studies, with follow‐up 1 week to 6 months after tamoxifen initiation for TT3 and 1 week to 13+ months for FT3. Total triiodothyronine was observed significantly increased compared to baseline before tamoxifen treatment in two studies^31^, ^39^ and significantly increased in present tamoxifen users versus ex‐tamoxifen users,^37^ while non‐significantly increased in another study.^35^ A statistically non‐significant decrease in TT3 was shown in two studies,^30^, ^41^ whereas unchanged TT3 was reported in one.^33^ Another study observed a significant decrease in FT3.^40^ Free triiodothyronine significantly decreased from baseline by a mean value of 12.7% at months 6 to 8 (*p* < 0.025) and by 14.5% after 13+ months (*p* < 0.025).

Changes in thyroid‐binding globulin: Four studies assessed TBG to elucidate the mechanism of the possible thyroid dysfunction. Thyroid‐binding globulin was assessed 3 to 6 months after tamoxifen initiation in three studies,^35^, ^38^, ^39^ but also beyond 6 months in one.^40^ Three studies reported significantly elevated TBG^38^, ^39^, ^40^ and one study observed the same trend of TBG elevation after 3 months.^35^ However, this study did not repeat the measurement after 6 months.^35^ Anker et al. were the only authors measuring TBG beyond 6 months.^40^ Tamoxifen treatment for up to 4 months did not cause TBG‐alteration, but TBG was elevated from baseline by a mean of 23.6% at months 6–8 (*p* < 0.001), by a mean of 22.2% after 9–12 months (*p* < 0.025) and furthermore by mean 26.4% after 13+ months of treatment (*p* < 0.001) compared to baseline/before initiation of tamoxifen.^40^

Thyroid dysfunction and weight gain: Bruning et al. showed that most patients treated with 10 mg tamoxifen in 6 months had a stable weight (mean 69.4 ± 10.3 kg in responding patients and 70.9 ± 7.6 kg in non‐responding patients), yet a general tendency to some, non‐specified weight gain has been observed in an unknown number of patients.^36^ In this study, all patients had a statistically significant increase in TT4 at month 2, and 35 patients at month 6 of tamoxifen treatment, whereas an unspecified number of patients had a statistically significant increase in TSH.^36^ In another study mean duration of 10 mg tamoxifen treatment was 19 months in patients where no other treatment than anti‐estrogen was used.^41^ Two patients had a non‐specified weight gain after 2 months of treatment, and both patients had elevated TSH, decreased TT3, and normal FT4, which were normalized after 5 months despite ongoing tamoxifen treatment.^41^

### 2.7 Patient characteristics and results in the letrozole group

Our review of the literature on letrozole treatment and thyroid function included five studies.^46^, ^47^, ^48^, ^49^, ^50^ No relevant studies of pre‐menopausal women have been found. All patients included had metastatic/locally advanced BC. Further information about these studies is shown in Table 2.

**TABLE 2:** Publications on letrozole and thyroid function included in the systematic review

Publication	Study design	Breast cancer stage	Mean/median age of patients (years)	Pre‐menopausal women (*n*)	Post‐menopausal women (*n*)	Prior breast cancer treatment / number of patients (*n*)	Dosis of letrozole (mg/day)	Duration of letrozole (months)	Time of thyroid hormones evaluation	Hormone measurements	Observed changes in thyroid function	Type of thyroid dysfunction
Bajetta E et al.^50^	Double‐blind randomized trial of two letrozole doses	Locally advanced and metastatic	Median 63	None	46	Tamoxifen, stopped at least 12 months before Some patients received chemotherapy	0.5–2.5	> 12	Baseline + months 1 + 3	TSH TT4 TT3	2.5 mg group at baseline: ↑TT4 ↑TSH →TT3 2.5 mg group at month 6: ↑TSH →TT3 0.5 mg group (baseline and month 6): →TT4 →TT3	Subclinical hypothyroidism Subclinical hypothyroidism None
Demers LM et al.^48^	Phase I, open label, dose‐range finding study	Metastatic	Unknown	None	8	Unknown	0.1–0.25	3	Unknown	Unknown	→ (data not shown)	None
Demers LM.^47^	Phase I, clinical efficacy study	Metastatic	Mean 56	None	23	Unknown	0.1–5.0	3	Baseline + biweekly for 12 weeks	TSH TT4 TT4 uptake	→TSH →TT4 →TT4 uptake	None
Iveson TJ et al.^46^	Phase I, open label, dose‐range finding study	Locally advanced and metastatic	Median 60	None	21	Tamoxifen (*n* = 21) Chemotherapy (*n* = 6)	0.1–2.5	1	Baseline + month 1	TSH	→TSH	None
Lipton A et al.^49^	Phase I, open label, dose‐range finding study	Metastatic	Median 61	None	19	At least one prior treatment Chemotherapy (*n* = 22) No systemic treatment at least 28 days before letrozole	0.1–5.0	3	Baseline + biweekly for 12 weeks	TSH TT4 TBG	→TSH →TT4 →TBG	None

*Note*: →: within reference range; ↑: above upper limit of normal.

Abbreviations: TBG, thyroxine‐binding globulin; TSH, thyroid stimulating hormone; TT3, total triiodothyronine; TT4, total thyroxine.

The studies were published from 1993 to 1999. Four studies were phase one studies or dose‐range finding studies.^46^, ^47^, ^48^, ^49^ Bajetta et al. have performed a double‐blinded, randomised clinical trial on two different letrozole doses.^50^ The median/mean age of the patients ranged from 39 to 81 years (Table 2). The median duration of letrozole therapy was 3 months, and the median dosage was 1.5 mg/day (range 0.1–5 mg/day) (Table 2). Thyroid hormones were evaluated in the median 3 months after initiation of letrozole treatment, whereas this was not specified in the study of Demers LM et al.^48^ In the same study, the authors did not specify which thyroid hormone measurements were performed.^48^ Most of the studies measured TSH, TT4, and TT3, and Lipton et al. measured also TBG^49^ (Table 2). None of the studies reported measurements of free thyroid hormones. Yet, in the study of Iveson et al., only TSH was measured.^46^ Only one study on letrozole mentioned weight gain.^50^

Changes in thyroid‐stimulating hormone: In 4 of 5 studies, TSH was measured from 2 weeks to 3 months after letrozole treatment was initiated.^46^, ^47^, ^49^, ^50^ No study reported a significant change in TSH after letrozole treatment. Bajetta et al. reported higher values of TSH at baseline and during the treatment with letrozole 2.5 mg/day in comparison with the low‐dosage regime (0.5 mg/day) but not significant over time in either treatment group.^50^

Changes in total thyroxine: TT4 was measured in 3 of 5 studies.^47^, ^49^, ^50^ Follow‐up time for TT4 after letrozole initiation was 2–12 weeks. Total thyroxine was not significantly changed, but Bajetta et al. observed a higher concentration of TT4 at baseline in the 2.5 mg/day patient group, which normalized during the follow‐up, while TT4 remained stable in the 0.5 mg/day patient group.^50^

Changes in total triiodothyronine/thyroid‐binding globulin: Only one study reported measurement of TT3 and TBG and no significant change was observed at 1 and 3 months follow‐up.^50^

Thyroid dysfunction and weight gain: Weight gain was reported in one out of 46 patients as an “adverse event” after more than 12 months of letrozole treatment (2.5 mg/day).^50^ In this study, no significant changes in thyroid hormone levels were observed, however, TSH at baseline and during the 2.5 mg/day letrozole treatment was higher than in patients treated with letrozole 0.5 mg/day.^50^

## 3 DISCUSSION

This systematic review summarizes the literature on the possible impact of anti‐oestrogens on thyroid function in pre‐ and postmenopausal breast cancer patients. Overall, the studies included in this review indicate that tamoxifen treatment is associated with an increased risk of thyroid dysfunction (i.e., subclinical hypothyroidism), which in some patients is being more pronounced with prolonged treatment. Studies of BC patients treated with letrozole do not show any clear indication of influence on thyroid function, however, thyroid hormones were not measured beyond 3 months of treatment. Only three studies informed on non‐specified weight gain,^36^, ^41^, ^50^ which could be related to changes in thyroid hormones after anti‐oestrogen treatment (statistically‐ and highly statistically significant increase in TSH, highly statistically significant increase in TT4), but other factors such as altered glucose and insulin metabolism are probably also of importance.^18^ A potential confounder related to weight gain is the use of birth control pills, however, oestrogen treatment is not indicated in BC patients taking anti‐oestrogens. Furthermore, a systematic review on the use of combination contraceptive pills did not observe a direct effect on weight.^54^

### 3.1 Tamoxifen and thyroid function

In total, 620 pre‐menopausal‐ and post‐menopausal patients receiving tamoxifen were included. In five studies (a total of 208 patients) tamoxifen caused a transient and mild thyroid dysfunction that is, subclinical hypothyroidism (statistically significant increase in TSH).^36^, ^39^, ^40^, ^41^, ^42^ The increase of TSH was most pronounced at months 2–12 of tamoxifen treatment in different dosages (10 mg/day–30 mg/day). Anker et al. suggested that tamoxifen may aggravate hypothyroidism in patients with subclinical thyroid disease, as observed changes in thyroid hormones are being more significant beyond 12 months of treatment.^40^ Few studies described an increase in TSH (statistically non‐significant),^30^, ^31^ while others did not show changes in TSH.^33^, ^35^, ^38^, ^43^ In the study of 59 pre‐menopausal women taking tamoxifen prevalence of amenorrhoea was 61%, and these patients had significantly lower TSH, yet within euthyroid ranges.^45^ The actual significance of this change is not known but requires attention due to the possible interaction of tamoxifen with the pituitary gland. In the studies describing a significant elevation of TT4 after 2–6 months, TSH was found to be unchanged^38^ or significantly increased.^36^, ^39^ Yet, TT4 was significantly decreased after 1 month of therapy together with an increase in TSH (statistically non‐significant).^30^ In five studies, FT4 was not influenced by tamoxifen,^32^, ^35^, ^39^, ^40^, ^41^ while only one study found significantly increased TT3 and non‐significantly increased FT4 and TSH at month 3 of tamoxifen treatment.^31^ Two studies mentioned weight gain after 6 months^36^ and after on average 19 months^41^ of tamoxifen treatment, respectively. In these studies, observed weight gain and thyroid dysfunction can be related, as indicated by a statistically significant increase in TSH. Grani et al. reported that the prevalence of hypothyroidism was twice as high as hyperthyroidism in 190 BC patients. However, the prevalence of autoimmune thyroid disease was almost 25%, influencing the thyroid hormone measurements.^44^ The immune therapeutics trastuzumab and pertuzumab are indicated in BC patients with HER2‐positive tumors. At present, six cases of trastuzumab‐associated autoimmune thyroid disease have been reported in the literature,^55^ the last one in 2021.^56^ No literature on pertuzumab and autoimmune thyroid disease has been found. However, Grani et al. did not report on the use of trastuzumab, which could influence the prevalence of autoimmune thyroiditis observed in their study.^44^ Although observed, all above‐described alterations of thyroid hormones seem to leave patients “clinically euthyroid”, yet no quality of life assessment has been done in any of the studies included in this review.

There are some limitations to consider in these studies. Firstly, the cancer staging is inhomogeneous—five studies did not specify it^31^, ^35^, ^37^, ^40^, ^44^ and other studies were performed in patients with different cancer types—from early‐stage to advanced BC, which could be influencing the occurrence of non‐thyroid illness. Secondly, patients were heterogeneous regarding menopause status, which was unknown in one study.^44^ Tamoxifen was used mostly in post‐menopausal women, although its primary indication in current guidelines is in pre‐menopausal women. Thirdly, follow‐up time on thyroid function vary—from 1 month to 5 years. Fourthly, studies have measured different thyroid parameters. One study measured only TSH^34^ and some authors measured other thyroid parameters, such as TT4, FT4, TT3, FT3, and TBG (Table 1). Finally, in some studies patients received radiotherapy/chemotherapy before tamoxifen,^31^, ^33^, ^40^, ^43^, ^45^ which could influence thyroid function. Not all studies have specified the previous cancer treatment.^36^, ^37^, ^38^, ^43^, ^44^ All together these limitations make solid conclusions hard to draw. In summary, the literature indicates that tamoxifen seems to influence thyroid function, posing a risk of subclinical hypothyroidism, which can be aggravated in some patients during a longer (>1 year) follow‐up of thyroid function measurements and can possibly be related to weight gain in some patients.

### 3.2 Possible mechanisms of thyroid dysfunction in tamoxifen‐treated patients

It is still speculative whether tamoxifen can influence TBG, the bioavailability of TT4/TT3, the hypothalamic–pituitary axis, or whether low plasma T3 concentrations in tamoxifen users may be influenced by non‐thyroid illness. As suggested, tamoxifen can suppress FT3 and FT4 and increase TBG,^40^ increase both TT4, TT3, and TBG,^35^ give only TBG elevation,^41^ or elevate both TT3, TT4, and TBG at the same time,^39^ illustrating a general inconsistency in the literature. Karami‐Tehrani et al. demonstrated that tamoxifen did not change the affinity of TT4 for TBG, probably due to the molecular size of tamoxifen being too large to enter the binding site.^57^ Other authors suggested the anti‐oestrogenic effect of tamoxifen on thyroid‐binding proteins due to a significant reduction of TT4.^33^ A study on 13 post‐menopausal tamoxifen‐treated women hypothesized that peripheral inhibition of TT3 and TT4 could be a mechanism for changes in thyroid parameters.^30^

Depending on the target tissue, tamoxifen can have different biological actions: predominantly anti‐oestrogenic effects in the breasts, yet oestrogenic effects in the uterus and the liver.^58^ Tamoxifen causes a mild increase in TBG concentrations in the liver,^58^ possibly as a result of an intrinsic, enhanced oestrogen‐agonistic effect.^36^, ^38^, ^59^ Mirzaei et al. speculated on tamoxifen bind to a protein with heavier glycoside than normal TBG, reducing TBG clearance and evoking TBG increase.^31^ It was suggested tamoxifen influences the synthesis of TT4 i.e. reduces TT4‐ and TT3 bioavailability, thus proposing thyroid dysfunction not to be related to TBG.^40^ For this reason, tamoxifen can aggravate pre‐existing subclinical thyroid disease, which is especially important in older patients. Other authors speculated on the interaction between tamoxifen and the hypothalamic–pituitary axis.^39^ They measured basal and TRH‐stimulated concentrations of TSH and TBG, which resulted in increased concentrations, suggesting the interaction of tamoxifen with T3‐receptor in the hypothalamic–pituitary axis.^39^ Acquired forms of TBG “deficiency” are worth mentioning, referring to an altered synthesis and/or degradation of TBG. These changes are observed in severe systemic illness and non‐thyroid illness, the latter probably being mediated by interleukin‐6, which seems to play a role by decreasing TBG gene transcriptional activity.^60^, ^61^ These mechanisms could also be a part of thyroid dysfunction observed in BC patients.

### 3.3 Letrozole and thyroid function

Five studies included in all 117 post‐menopausal BC patients on letrozole treatment and thyroid function are included in this systematic review.^46^, ^47^, ^48^, ^49^, ^50^ Generally, letrozole did not cause statistically significant changes in thyroid hormones. In addition, Lipton et al. did not observe any statistically significant changes in TBG at month 3 of follow‐up.^49^ Although Bajetta et al. reported higher values of TT4 and TSH in the 2.5 mg patient group, no significant change over time was registered.^50^ All patients in letrozole studies remained euthyroid, but one study described a patient on 2.5 mg/day of letrozole treatment experiencing weight gain, whose baseline TSH and TT4 were higher than patients in the low‐dosage group.^50^

There are some limitations to consider in these studies. Firstly, thyroid hormone measurements were performed after 3–12 months of letrozole therapy, so long‐term side‐effect cannot be discussed. Secondly, in the majority of studies patients were pre‐treated with one or more cancer treatments (chemotherapy, radiation therapy, tamoxifen, etc.), which could influence the study results.^46^, ^49^, ^50^ Thirdly, only one study measured TBG.^49^ In another study, only TSH was measured,^46^ whereas Demers et al. did not specify which thyroid parameters were measured but concluded that thyroid function was “not compromised”.^48^

## 4 CONCLUSIONS

In conclusion, based on current literature, tamoxifen, but not letrozole treatment provides a risk of subclinical hypothyroidism during the first year of treatment in patients with BC. Most of the studies published on tamoxifen document some degree of increase in TSH without a significant decrease in peripheral hormones, namely subclinical hypothyroidism. It is yet unknown whether this effect on thyroid hormones continues or is being more pronounced if patients are followed during more than 1 year of treatment. A single study indicates further significant changes in thyroid hormones beyond 1 year of tamoxifen treatment. If anti‐oestrogens affect thyroid function, it might partly explain why some BC patients experience weight gain. However, other factors, such as how cancer treatments affect body composition, plasma lipids, or insulin sensitivity, should also be considered. Although several more well‐powered studies are needed to evaluate thyroid function after long‐term letrozole treatment, literature data from this systematic review suggest increased awareness of thyroid function in patients treated with tamoxifen. Most of the studies performed on thyroid function and anti‐oestrogen therapy are observational cohort studies, and more prospective studies with longer follow‐up measurements of thyroid hormones are preferable.

## AUTHOR CONTRIBUTION

Djordje Marina performed the search, selected the articles for systematic review, performed the risk of bias, and wrote the paper; Åse Krogh‐Rasmussen contributed to the writing of the paper and was especially focused on the part with thyroid function; Michael Andersson contributed to the writing of the paper and was especially focused on the oncology part of the paper; Kristian Buch‐Larsen performed the search and the risk of bias and contributed to the writing of the paper; Linn Gillberg contributed to the writing of the paper; Peter Schwarz initiated the study and supervised all aspects of the process as well as contributed on the writing of the paper.

## FUNDING INFORMATION

Funding was received from Svend Andersen Foundation, Aase and Ejnar Danielsens Foundation, and the Novo Nordisk Foundation. Neither had a role in writing the manuscript or the decision to submit it for publication.

## CONFLICT OF INTEREST

No potential conflict of interest was reported by the author(s).

## ETHICS STATEMENT

Ethical approval was not needed for this systematic review.

## Supporting information


Appendix S1

Appendix S2

## Data Availability

Data sharing is not applicable to this article as no new data were created or analyzed in this study.
