# Supplementary figures and images for: Influence of the anti‐oestrogens tamoxifen and letrozole on thyroid function in women with early and advanced breast cancer: A systematic review
Source: Cancer Med. 2022 Jun 24;12(2):967–82. doi: 10.1002/cam4.4949 (PMC9883413; doi:10.1002/cam4.4949)

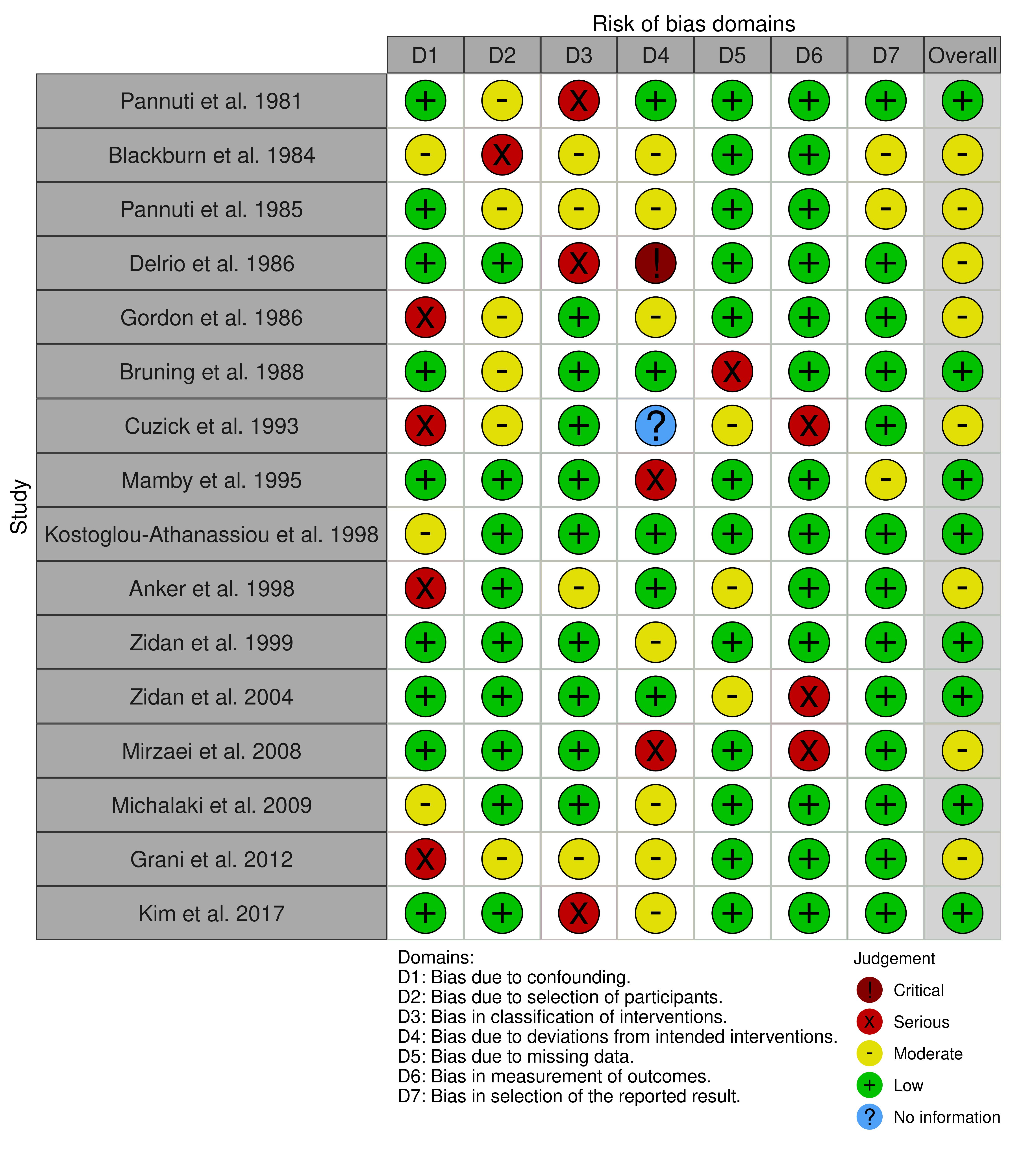

Supplement: Supplementary file 1 — Appendix S1 [file CAM4-12-967-s002.jpeg]

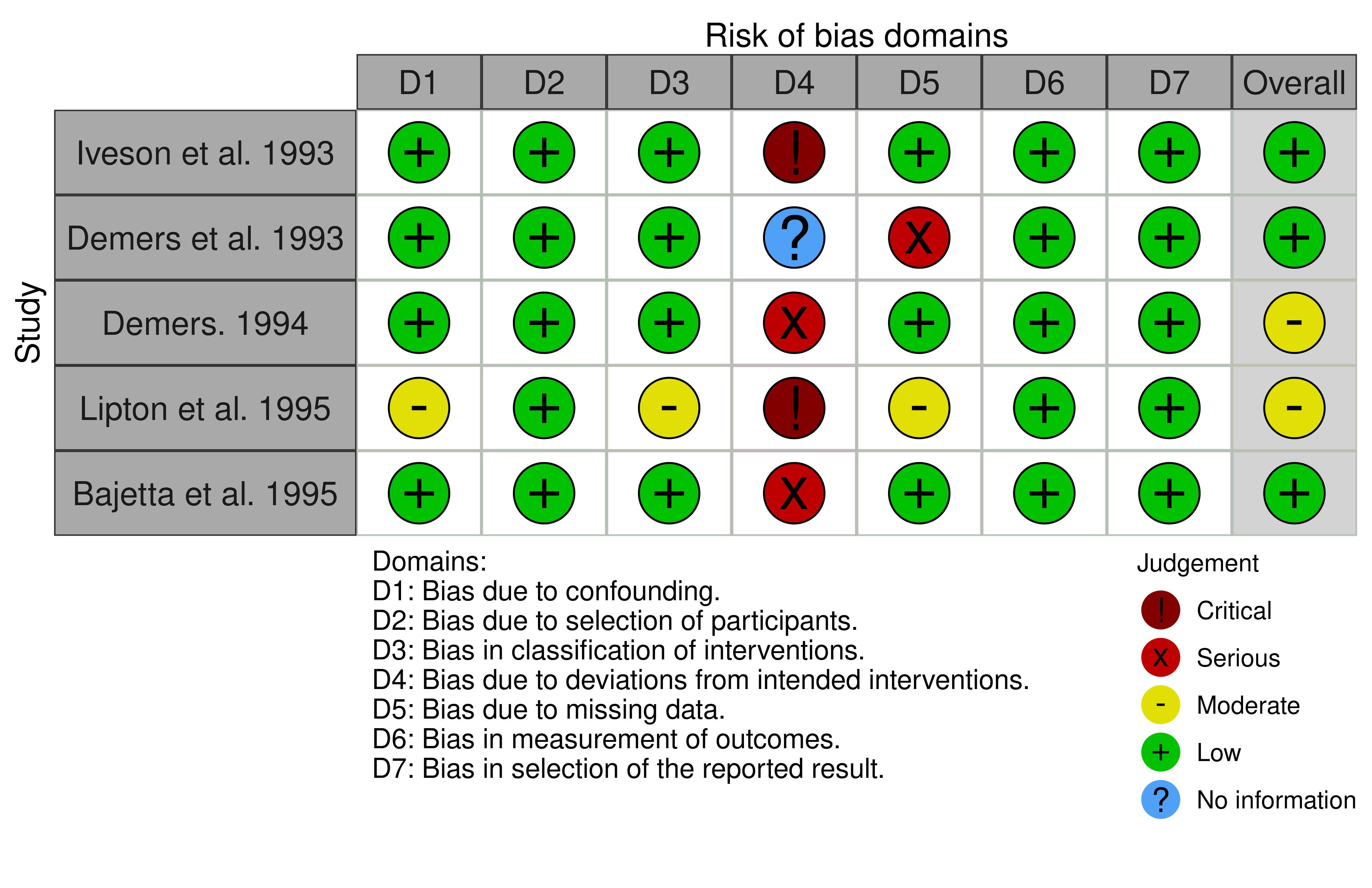

Supplement: Supplementary file 2 — Appendix S2 [file CAM4-12-967-s001.jpeg]
